# A Mathematical-Biological Joint Effort to Investigate the Tumor-Initiating Ability of Cancer Stem Cells

**DOI:** 10.1371/journal.pone.0106193

**Published:** 2014-09-03

**Authors:** Chiara Fornari, Marco Beccuti, Stefania Lanzardo, Laura Conti, Gianfranco Balbo, Federica Cavallo, Raffaele A. Calogero, Francesca Cordero

**Affiliations:** 1 Department of Computer Science, University of Torino, Torino, Italy; 2 Department of Molecular Biotechnology and Health Sciences, Molecular Biotechnology Center, University of Torino, Torino, Italy; Cleveland Clinic, United States of America

## Abstract

The involvement of Cancer Stem Cells (CSCs) in tumor progression and tumor recurrence is one of the most studied subjects in current cancer research. The CSC hypothesis states that cancer cell populations are characterized by a hierarchical structure that affects cancer progression. Due to the complex dynamics involving CSCs and the other cancer cell subpopulations, a robust theory explaining their action has not been established yet. Some indications can be obtained by combining mathematical modeling and experimental data to understand tumor dynamics and to generate new experimental hypotheses. Here, we present a model describing the initial phase of ErbB2^+^ mammary cancer progression, which arises from a joint effort combing mathematical modeling and cancer biology. The proposed model represents a new approach to investigate the CSC-driven tumorigenesis and to analyze the relations among crucial events involving cancer cell subpopulations. Using *in vivo* and *in vitro* data we tuned the model to reproduce the initial dynamics of cancer growth, and we used its solution to characterize observed cancer progression with respect to mutual CSC and progenitor cell variation. The model was also used to investigate which association occurs among cell phenotypes when specific cell markers are considered. Finally, we found various correlations among model parameters which cannot be directly inferred from the available biological data and these dependencies were used to characterize the dynamics of cancer subpopulations during the initial phase of ErbB2^+^ mammary cancer progression.

## Introduction

There is increasing evidence to support the theory that the progression of many human tumors is controlled by a cellular hierarchy in which Cancer Stem Cells (CSCs) constitute the core of tumor mass. This hierarchical organization is due to CSC properties, such as strong tumorigenic capacity, self-renewal, and differentiation into non-stem cells [1]. Specifically, CSCs can proliferate either symmetrically or asymmetrically. In the former case two daughter cells with CSC features are generated, in the latter one a multipotent Progenitor Cell (PC) and a CSC-like daughter cell are produced. PCs proliferate giving rise to daughter cells which are more differentiated and endowed with a lower proliferative potential than their mother cells. Hence, this mechanism leads to heterogeneous cancer subpopulations characterized by a high degree of differentiation and a loss of proliferation ability [2]. The CSC biology has been extensively studied in the last few years [3]–[6] but it is not fully understood yet. Many crucial issues are still under investigation, such as dynamics related to the initial phase of tumor growth [7]. In this complex context, experimental studies *per se* may be infeasible (both from budget and time point of views) to investigate all possible combinations of the crucial factors that regulate tumor onset and development. Therefore, the contribution of mathematical modeling in cancer biology can be useful in order to restrict the number of wet-lab experiments needed for testing hypotheses and for generating new conjectures. Indeed, the idea underlying the definition and analysis of a model is to identify the macro events characterizing the biological phenomena under study.

Several in-silico models describing cancer cell population dynamics have contributed to an improved characterization of tumor progression. In particular, Molina-Pena and Álvarez built a flexible deterministic model proving that there are some common kinetic features of tumor growth among different cancers, consistent with the CSC hypothesis [8]. Marciniak-Czochra's group has mathematically investigated the role of CSC symmetrical proliferation with respect to tumor maintenance and published their results in several papers [9]–[11].

In other papers, the study of cell population dynamics in specific tissues has been reported, emphasizing the role of CSC-based organization in growth and regeneration processes. Johnston *et al.* have characterized population dynamics in healthy crypts, demonstrating that changing any of the key parameters can initiate cancerogenesis [12],[13]. Compartment models on colonic crypts have also been proposed by Tomlinson *et al* [14],[15] and by Mirams *et al*, the latter of which investigated how space influences cell dynamics in colonic crypts [16].

Lander's group studied the general process of tissue development and regeneration, where impressive examples of tight control of cell growth and differentiation can be found [17]–[21]. They investigated also control mechanisms in tumor progression showing that cancer growth is controlled by the spatio-temporal dynamics of key signaling processes, expressed as positive and negative feedback loops [22].

Lastly, Michor and coworkers have proposed several models describing the tumor initiation and progression, starting from the clonal evolution theory [23]–[26]. Their works contributed to the characterization of the fundamental principles governing dynamics of oncogene activation and tumor-suppressor inhibition [23].

The aim of our work is to study the tumorigenic capability of CSCs using an integrated approach where a mathematical model describing the initial phase of cancer progression has been constructed and calibrated by exploiting data coming from *in vivo* and *in vitro* experiments.

Even though it is well known that breast cancer is composed of heterogeneous cancer cell subpopulations organized in a hierarchical manner, the dynamics regulating proliferation, death, and differentiation of CSCs and progenies are difficult to infer from tumor volume data alone.

Here, we present a study on ErbB2^+^ mammary cancer through the synergistic union of wet-lab experiments and applied mathematical techniques. We use CSC theory to define a system of Ordinary Differential Equations (ODEs) describing the initial phase of cancer progression. We refer to this model as *essential* in order to focus the attention on its basic, but not simplistic form: it provides a system abstraction which is relatively simple, but still able to capture the key aspects of breast cancer. Moreover, quantitative and qualitative analysis of this ODE system has been performed to highlight the relations among proliferation, death, and differentiation rates which cannot be directly inferred from biological experiments.

This mathematical model has been elicited from several papers describing CSC evolution [2],[27],[28], and from experimental evidences [29]. Indeed, in [29] we have previously shown that mammary cancers which spontaneously arise in BALB-neuT mice - transgenic for the activated rat ErbB2 oncogene - contain a population of CSCs able to generate mammospheres *in vitro*, which are also endowed with the ability to initiate tumors *in vivo*. In the same paper, we also reported that mammospheres obtained from an epithelial cell line derived from a BALB-neuT carcinoma, named TUBO cells, express markers associated with CSC phenotype. Moreover, TUBO cells are able to efficiently generate tumors when implanted subcutaneously (s.c.) into syngeneic mice [30]. Summarizing, starting from an essential description of breast cancer dynamics, we calibrated our model by considering several experimental conditions, and we extrapolated relations among the critical parameters hence inferring the rules which control the initial cancer progression observed in mice. Moreover, we used the model to investigate the distribution of known cell markers among the various tumor cell populations, and to design new biological experiments for the CSC characterization.

## Material and Methods

### Biological Experiments

#### Cell and mammosphere cultures

TUBO epithelial cells (an ErbB2^+^ cloned cell line established from a mammary carcinoma arising in a BALB-neuT female mouse [31],[32]) were cultured in DMEM supplemented with 20% FBS. To generate non-adherent spherical clusters of cells (mammospheres), TUBO cells were detached and plated in ultra-low attachment flasks (Sigma-aldrich) at 6×10^4^ viable cells/ml in mammosphere medium. This medium consists of serum-free DMEM-F12 medium (Invitrogen Corp.) supplemented with 20 ng/ml basic Fibroblast Growth Factor (bFGF), 20 ng/ml Epidermal Growth Factor (EGF), 5 microg/ml insulin, and 0.4% bovine serum albumin (BSA) - all from Sigma-Aldrich [30]. Mammospheres named P1 were collected after 7 days and disaggregated using enzymatic and mechanical dissociation. P1-derived single-cell suspensions were seeded again at 6×10^4^ viable cells/ml to generate new mammospheres, named P2. The process was repeated a third time to generate P3.

#### Mouse model

Female BALB/c mice (Charles River Laboratories) were maintained at the Molecular Biotechnology Center of the University of Torino and treated in accordance with the University Ethical Committee and European guidelines. All *in vivo* experiments were approved by the University of Torino Ethical Committee and by the *Italian Health Department* (Rome, Italy). TUBO (10^3^ and 10^5^) and P3 (10^3^) cells were implanted s.c. into the left flanks of BALB/c mice. Mice were killed according to the ethic protocol when the average of the two perpendicular diameters exceeded 10 mm. The growth of tumors related to these three different initial conditions was monitored every week and reported as average diameter (mm). Let us note that the three initial conditions - in terms of cell types and concentrations - lead to three sets of experiments that will be referred in the rest of this paper using the notation *exp1*, *exp2*, and *exp3*.

#### FACS analysis

After 7 days of culture, TUBO, P1, P2, and P3 cells were collected and disaggregated using enzymatic and mechanical dissociation. Then they were washed in PBS (Sigma-Aldrich) supplemented with 0.2% BSA and 0.01% sodium azide (Sigma-Aldrich), and stained for membrane antigens. The following antibodies were used: (i) Alexa Fluor647-conjugated anti-Stem Cell Antigen-1 (Sca-1), (ii) PE-conjugated anti-CD44 and PE/Cy7-conjugated anti-CD24 (all from Biolegend). All samples were collected and analyzed using a CyAn ADP Flow Cytometer and Summit 4.3 software (DakoCytomation).

### Mathematical approach

The above biological data were integrated in a mathematical framework to reproduce the observed tumor growth and to infer further knowledge on the relations occurring among the crucial events involving cancer cell subpopulations. In detail, our mathematical approach consists of the following main steps:

(i) tumor growth rates were estimated by fitting measured volume data with the Malthusian model - *Malthusian growth model* subsection;

(ii) volume growth and subpopulation dynamics were described by a system of differential equations defined from the assumptions of CSC theory - *Breast cancer compartment model* subsection;

(iii) the model solution was analytically evaluated to establish the temporal evolutions of the system and to find the parameters responsible for tumor progression - *Model solution* subsection;

(iv) an aggregation process was performed on model parameters to define new parameters which refer to groups of similar cellular events. This aggregation process resulted in a first reduction of the parameter space. Some biological constraints were introduced to make the model consistent with experimental data and properties reported in the literature. This led to a further reduction of the parameter space - *Parameter settings* subsection;

(v) volume data were fitted with the proposed model, from which cell subpopulation dynamics were also derived. These results, which turned out to be consistent with both the tumor growth data and the imposed properties, where then used as a starting point for further analyses on model parameters. Specifically, some hidden relationships among cellular events were discovered, so that the role of CSCs in cancer progression was better characterized - *Data fitting* subsection.

Technical details about each of these steps are reported in the following sections and in Supplementary Material.

#### Malthusian growth model

Tumor growth can be conveniently described by means of the Malthusian model [33] assuming that there are no nutrient transport limitations and that space constraints are not significant. The Malthusian growth model, also called the power-law model, describes an exponential growth based on a constant rate 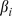 through the equation:
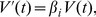
(1)where *i*  = *exp1*, *exp2*, and *exp3*; see the *Mice model* subsection. Note that this assumption is reasonable since the hypothesis that cancer volume *V*(*t*) increases with a constant cellular growth rate is mostly acceptable during its initial progression phase.

#### Breast cancer compartment model

Even though the Malthusian growth model gives a good representation of the overall tumor growth, it is not able to directly capture relationships among different cancer cell subpopulations. Thus, to point out which are the key factors in tumor progression, we represented cell subpopulation dynamics using the following system of linear ODEs:
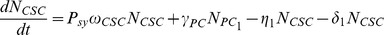


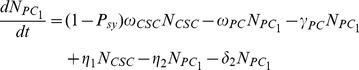


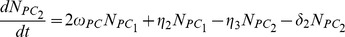


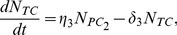
(2)where 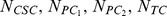 are the numbers of CSCs, PCs_1_, PCs_2_, TCs, respectively.

This system was designed taking inspiration from the model reported in the work [27] and then integrated with knowledge about cancer dynamics derived from several papers, among which [2],[8],[34]. Our model takes into account the self-renewing ability of CSCs that can be symmetrical (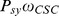) or asymmetrical (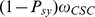). Moreover, a progression of CSCs - called CSC commitment (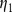) - can occur in terms of differentiation when a CSC gives rise to a multi-potent PC. Equations in (2) model two layers of PC subpopulations: PCs_1_ and PCs_2_. The first one is characterized by proliferation and differentiation capabilities that are both involved into the progression of PCs_2_ which develop into non-proliferative Terminally differentiated Cells (TCs). We considered also the de-differentiation (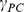) of PCs_1_ into CSCs, as described in [35] and mathematically characterized in [36]. Lastly, cancer stem, progenitors and differentiated cells are affected by a death rate (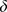) specific for each cell type.

The system of ODEs represented by Equations (2), augmented with the following set of initial conditions
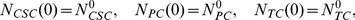
(3)constitutes a Cauchy problem which describes the temporal evolution of breast cancer, with a focus on its different cell subpopulations.

#### Model solution

It is well known that a Cauchy problem of the type represented by Equations (2) and (3) can be analytically solved to obtain the size of each cell subpopulation at any time point [37]. Specifically, the model solution is derived from the model eigensystem which determines the temporal evolution of the system and its stability as well [37]. In particular, among all these eigenvalues, there is one called growth constant (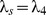) which defines the system growth rate; its corresponding eigenvector (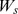) defines the system growth direction.

To explore model (2) from different perspectives, we have performed a set of qualitative and quantitative analyses. Results coming from these two analyses are complementary, and contribute to obtain a global and complete understanding of the model. More details on the model solution and on how the model eigensystem controls the system behavior are provided in Equations (s.3) of Supplementary Material.

#### Parameter settings

**Parameter aggregation.** The model described by system (2) comprises four independent variables, i.e. one for each cell subpopulation, and ten parameters defining cell dynamics. More precisely, each parameter describes a specific cellular event (proliferation, differentiation,…) and it is independent of the others. This high *specificity* provides a complete description of the subpopulation dynamics, but it requires a high number of parameters difficult to estimate. To cope with this, we have defined a new set of *aggregated* parameters grouping the original kinetic parameters as follows:
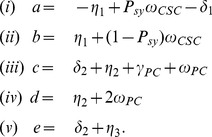
(4)


This *aggregating process* provided new parameters describing the flow of each cell subpopulations in the model. The meaning of these new parameters can be explained by the following biological interpretation: (i) *a* expresses CSC variation, neglecting the de-differentiation term 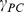; (ii) *b* describes the increasing rate of PCs_1_; (iii) *c* represents the PC_1_ decreasing rate; (iv) *d* is the increasing rate of PCs_2_; and (v) *e* is the decreasing rate of PCs_2_. Let us note that this aggregation has been a crucial step of the analysis process for several reasons. It decreased the complexity of the ODE system reducing the dimension of the parameter space, and it made equations easier to manage. Using the aggregate parameters (4) in system (2), we decrease the number of parameters that must be inferred from experimental data, being *a*, *b*, *c*, *d*, *e*, 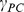, 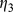, and 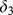 the only ones that had to be estimated. Moreover, from equations (4), it is clear that all aggregate parameters are positive except *a*, whose sign depends on the balance among CSC symmetrical proliferation, differentiation, and death rate.

**Parameter space is restricted by biological constraints.** To make the model behavior consistent with the biological phenomenon under investigation, we imposed a set of constraints on the parameter values. Part of them are related to biological knowledge on breast cancer growth, while others derive from our experimental data. From the evaluation of tumor growth in BALB/c mice we used the Malthusian model as a first approximation of cancer progression which allowed to estimate the experimental growth rates 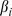 (with *i* =  *exp1*, *exp2*, *exp3*) from the available data. Then, the growth rate 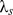 in the linear ODE system was set equal to 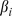. The progenitor and terminal subpopulations represent the majority of cancer cells [28]; however, the proportion of all subpopulations should be determined by the type and number of cells injected in the mice. From our experiments we deduced that P3 cells are more enriched in CSCs than TUBO cells. In particular, the analysis of Sca-1^+^ and CD44^+^/CD24^−^ cells revealed the CSC amount in each mammospheres passages.

During the “exponential growth phase” the ratios between the subpopulation sizes and total cell number (*N_TOT_*) are functions of time which became practically constant as the time parameter grows large [38]–[40]. Therefore, we imposed the following conditions on the cell subpopulation fractions:
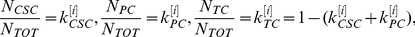
(5)where *i* =  *exp1*, *exp2*, *exp3* to reproduce each type of cell injection. Note that, knowing the analytic solution of model (2), above conditions (5) can be easily expressed using system eigenvectors, as reported in Supplementary Material - Equations (s.7).

At last, Tang [2] describes the de-differentiation as a rare event since it occurs only under particular conditions, the variation interval of 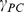 - defined in the data fitting process - was chosen smaller than those of the other parameters.

To conclude, taking into consideration all previous constraints, the number of free parameters was reduced and the parameter space was downsized since 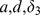 and 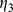 were directly inferred from experimental data, while *b*, *c*, *e* and 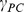 had to be computed considering all their possible positive values.

#### Data fitting

System (2) describes how the total number of breast cancer cells - and the corresponding tumor volume - change during time. Assuming that each spherical shaped cell gives the same contribution to the spherical tumor, we stated that a tumor grows proportionally to the total number *N_TOT_* of cells. Numerically, we had 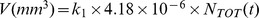, where *k*_1_ is a volume-growth constant, which accounts for the percentage of quiescent/dead cells in tumor, i.e. a new parameter.

The parameter space was explored using the standard Minimum Least Square (MLS) technique to produce the best fit of breast cancer data. This method searches the parameter combination that minimizes the sum of squared residuals. Note that the MLS algorithm searches the optimal solution, within the parameters space, starting from a set of fixed values 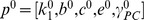. To find the best data fitting, we run the MLS method several times, using different initial parameter choices. These starting values were defined through the latin hypercube sampling technique [41] using the following distributions: (i) 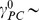 Unif 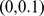; (ii) 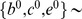 Unif (0, 5). The variation intervals were chosen in accordance with the literature and the range of 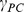 was set smaller than that of other parameters as mentioned before. Finally, let us point out that there are several types of distributions that can be used as probability density functions to define the starting points of the method. This choice should depend on a priori information but, when no data are available, the natural assumption is the uniform distribution.

## Results

### Cancer growth model

**Figure 1:**
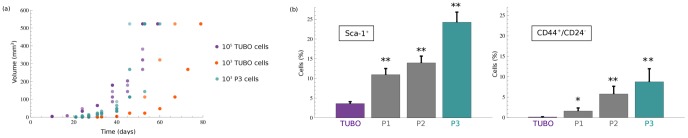
Tumors growth data and stem cell markers expression. Panel (a): tumor onset ability of 10^5^ TUBO cells (violet points), 10^3^ TUBO cells (orange points) and 10^3^ P3-derived cells (blue), injected in mice. Panel (b): Sca-1^+^ and CD44^+^/CD24^−^ histograms reporting the mean ± SEM of positive cells, from six independent experiments. *p<0.1, **p<0.05, Wilcoxon test.

The *in vitro* experiments generated three passages of mammospheres enriched in CSCs starting from a single cell suspension of TUBO cells. In detail, floating spherical mammospheres developed (P1) after a 2 day culture and became symmetrically encapsulated after 7 days to form *golf ball*-like structures that afterward got to be hollow inside around the third week and did not grow or expand further. These P1 mammospheres were dissociated after a culture of 7 days and propagated in secondary (P2) and tertiary (P3) sphere passages. Clones generated from TUBO, P1, P2 and P3 cells were counted in order to weigh up the *in vitro* self-renewal potential of mammospheres. To determine the tumorigenic potential of mammospheres with respect to TUBO cells, we selected three initial cell concentrations: 10^5^ TUBO, 10^3^ TUBO, 10^3^ P3 cells, and we implanted them s.c. in syngeneic BALB/c mice. Injection of 10^3^ P3-derived cells gave rise to fast growing tumors in all mice, whereas a similar challenge of 10^3^ TUBO cells gave rise to tumors in 4 out of 6 mice, but only two tumors reached a 10 mm average diameter in the following 100 days. In detail, the percentage of tumor takes in mice injected with 10^5^ TUBO or 10^3^ P3-derived cells was 100%, while this value decreased to 67% in mice injected with 10^3^ TUBO cells. Let us note that for further analysis we considered only those mice in which cancer grew exponentially, see Table S1. The higher propensity to form breast cancer in 60 days of 10^5^ TUBO cells and 10^3^ P3 with respect to 10^3^ TUBO cells can be appreciated in Figure 1 (panel a).

The Malthusian model (1) was fit to measured cancer growth data to determine the bulk growth parameters (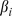) for each cancer scenario - i.e. 10^3^, 10^5^ TUBO cells, and 10^3^ P3-derived cells. Moreover, these numerical growth rate estimations confirmed the experimental evidence that P3 cells have a larger tumorigenic potential than any concentration of TUBO cells, when injected in mice. Indeed, in 10^3^ TUBO scenario 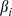 is equal to 0.06, in 10^5^ TUBO scenario 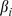 is equal to 0.07, and in 10^3^ P3 scenario 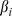 is equal to 0.09. The model curve-fit provided by the Malthusian model is reported in Figure S1. As we already observed, even though this model accurately describes cancer growth in terms of volume expansion, it does not characterize the relations among cancer cell subpopulations.

We inferred CSC, PC and TC behaviors by means of the *essential* model (2) that includes the cell subpopulation distributions in tumor mass starting from the assumptions of CSC theory. Then, we tuned the aggregated parameters using the biological constrains, described in Material and Methods, combined with the experimental values of breast cancer volumes and the proportion of CSCs derived from the percentage of Sca-1^+^ and CD44^+^/CD24^−^ cells. A FACS analysis of stem cell markers showed that Sca-1 [30] is barely expressed on TUBO cells while its expression progressively increases from P1 to P3-derived cells. The CSC enrichment in mammosphere passages was further confirmed by the progressive increase of CD44^+^/CD24^−^ cells observed from TUBO to P3 mammospheres, as reported in Figure 1 (panel b).

**Figure 2:**
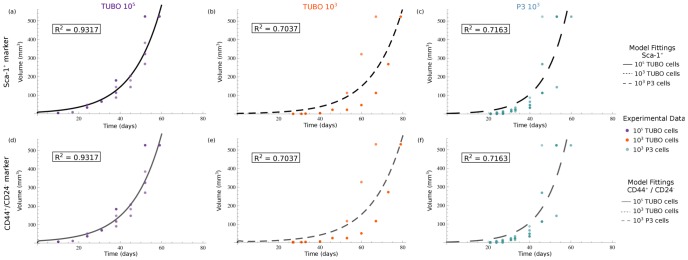
The breast cancer compartment model fittings. Each panel reports a comparison between experimental volumes (points) and the best model-fits (lines), considering a specific cell injection (10^5^ TUBO, 10^3^ TUBO or 10^3^ P3) and a fixed CSC concentration (given by Sca-1^+^ or CD44^+^/CD24^−^ cells). In detail: panels (a), (b), (c) use Sca-1^+^ proportions and correspond to 10^5^ TUBO, 10^3^ TUBO and 10^3^ P3 cells injections, respectively. On the other hand, in panels (d), (e), (f) are reported results obtained from 10^5^ TUBO, 10^3^ TUBO and 10^3^ P3 cells injections, using cells proportions defined by CD44^+^/CD24^−^ cells. For each plot, model parameters are those reported in Table 1.

For each initial condition, we performed a number of MLS runs greater than 10 and, among the results provided by these runs, we selected the *best-fit* which minimizes the sum of squared residuals. Notice that different initial conditions were determined by the type and number of cell injected (*exp*_1_, *exp*_2_, *exp*_3_) and by the stem marker used to quantify CSC proportion (Sca-1^+^ or CD44^+^/CD24^−^). The best-fit parameters estimated for each of these initial conditions are reported in Table 1, while Figure 2 shows obtained fits. The same volume-data (i.e. those of Figure 1, panel A) were fitted by the model when either Sca-1^+^ or CD44^+^/CD24^−^ proportions were assumed to infer CSC percentage within the tumor mass. More precisely, the volume-data arising from the same cell injection (*exp*_1_, *exp*_2_, *exp*_3_) were used twice: one for each marker considered. Specifically, in Figure 2, panels a, b, c show the model curve-fitting for each initial cell concentration, considering cell subpopulation proportions extrapolated from Sca-1^+^ data; while panels d, e, f show the model fitting when cell proportions are obtained considering CD44^+^/CD24^−^ cells. As reported by Figure 2, the different fitting curves were equivalent in terms of the produced error. However, subpopulation dynamics changed when different proportions were assumed as shown by Figure S2 for the injection of 10^3^ TUBO cells.

**Table 1:** Best-fit parameters.

	Exp.	R^2^	kl	b	c	e	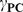	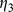	d	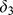	a	R_0_
**Sca-1^+^**	10^5^ TUBO	0.9318	14.53	1.96	2.24	5.14	0.81	4.33	25.48	0.81	−0.62	0.088
	10^3^ TUBO	0.7038	367.65	0.83	1.24	4.41	0.67	3.69	30.71	0.72	−0.36	0.088
	10^3^ P3	0.7164	657.02	0.61	2.17	0.51	0.02	0.39	1.95	0.11	0.09	0.095
**CD44^+^/CD24** ^−^	10^5^ TUBO	0.9318	1.67	4.37	2.68	3.34	0.40	2.73	423.28	0.60	−0.57	0.082
	10^3^ TUBO	0.7038	141.59	3.20	2.08	2.67	0.08	2.20	362.12	0.48	−0.05	0.073
	10^3^ P3	0.7164	647.77	0.39	0.99	0.93	0.04	0.88	3.90	0.05	0.08	0.095

Parameter values which minimize the least square error, i.e. the sum of the squares residuals, are reported. Each row corresponds to a specific initial condition and cells proportions experiment. Column *R*_0_ specifies which is the value of the reproduction rate (R_0_) of CSCs in each best fit. As defined by Eq. (6), this parameter has to be positive in order to observe a tumor exponential growth.

### How CSCs (mathematically) affect the tumor growth

Temporal evolutions predicted by the *essential* model were analytically determined studying its eigensystem. Specifically, in Material and Methods and Supplementary Material we pointed out how the growth constant (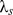) and its correspondent eigenvector (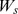) can be used to determine the system growth rate and direction. Explicit expressions of eigenvectors, as well as a discussion on their signs (see Figure S3), are reported in Supplementary Material. Summarizing, from this study we found that the stability of system (2) is controlled by the eigenvector 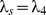.

To biologically characterize this result, we have defined the reproduction rate *R*_0_ of CSCs as their variation rate due to *intra*-CSC mechanisms plus the rate of PCs_1_ which undergo de-differentiation, namely 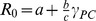. Therefore, the equilibrium conditions can be expressed as:
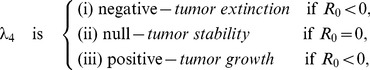
(6)similarly to epidemiological studies [42]. Notice that this result is in line with the current knowledge on the kinetic of CSC-based models which point out the role of CSC and PC_1_ as tumor driving force [9],[12],[43], considering also cell migration as reported in [44],[45]. Indeed, Equations (6) emphasize how three possible tumor-scenarios depend only on CSC reproduction rate, thus remarking the central role of these cells in tumor progression. In particular: if (i) is satisfied, the system moves toward extinction (asymptotic stability), i.e. there is no tumor establishment; when condition (ii) occurs, the model reaches a steady state, i.e. tumor grows until it stabilizes to a plateau (cell homeostasis); while when (iii) is met, the system grows exponentially, i.e. there is an unbounded tumor growth. Let us note that the trivial steady state (i.e. cell homeostasis) is very sensitive to small changes in combined CSC and PC_1_ variation rates, since it occurs only when *R*_0_ = 0. On the other hand, cell exponential growth (*R*_0_>0) and tumor extinction (*R*_0_<0) are more robust with respect to such variations. Therefore, despite this *structural instability* typical of linear systems, we opted for a linear model able to well reproduce the tumor exponential growth observed in mice. Indeed, at this stage of our study, we were interested in describing breast cancer growth during its initial exponential phase.

**Figure 3:**
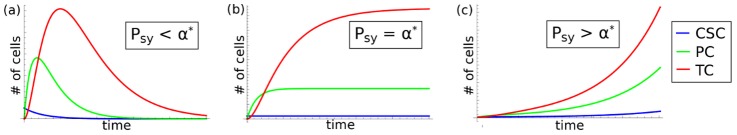
Three possible tumor scenarios. Each panel shows a possible system behavior: (a) 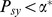, corresponding to extinction; (b) 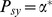, population move towards steady state; (c) 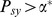, population grow exponentially.

Lastly, being 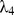 defined in terms of the disaggregated parameters (see Equation (s.5)) it is possible to derive a new set of inequalities related to those of Equations (6). Note that CSC symmetrical proliferation probability (*P_sy_*) expresses the variability in CSC division regulating the system stochasticity; indeed, different system behaviors can arise varying *P_sy_*. To quantify the threshold associated with these behaviors, we solved the inequalities (6) with respect to *P_sy_* and we grouped all other terms in the new variable 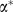. Specifically, solving systems (4) and (6) we obtained the following set of relations:
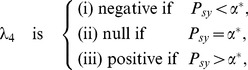
(7)which agree with the well-known central role of CSC symmetrical proliferation in cancer evolution [11]. The threshold 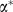, whose complete expression is
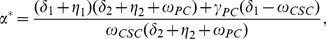
(8)represents a critical value which discriminates among possible tumor evolutionary-scenarios, as shown in Figure 3. 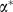 involves many parameters, suggesting that tumor evolution can be influenced acting on many of these parameters, i.e., on many of their corresponding cellular events.

### Discovering relationships among parameters

**Figure 4:**
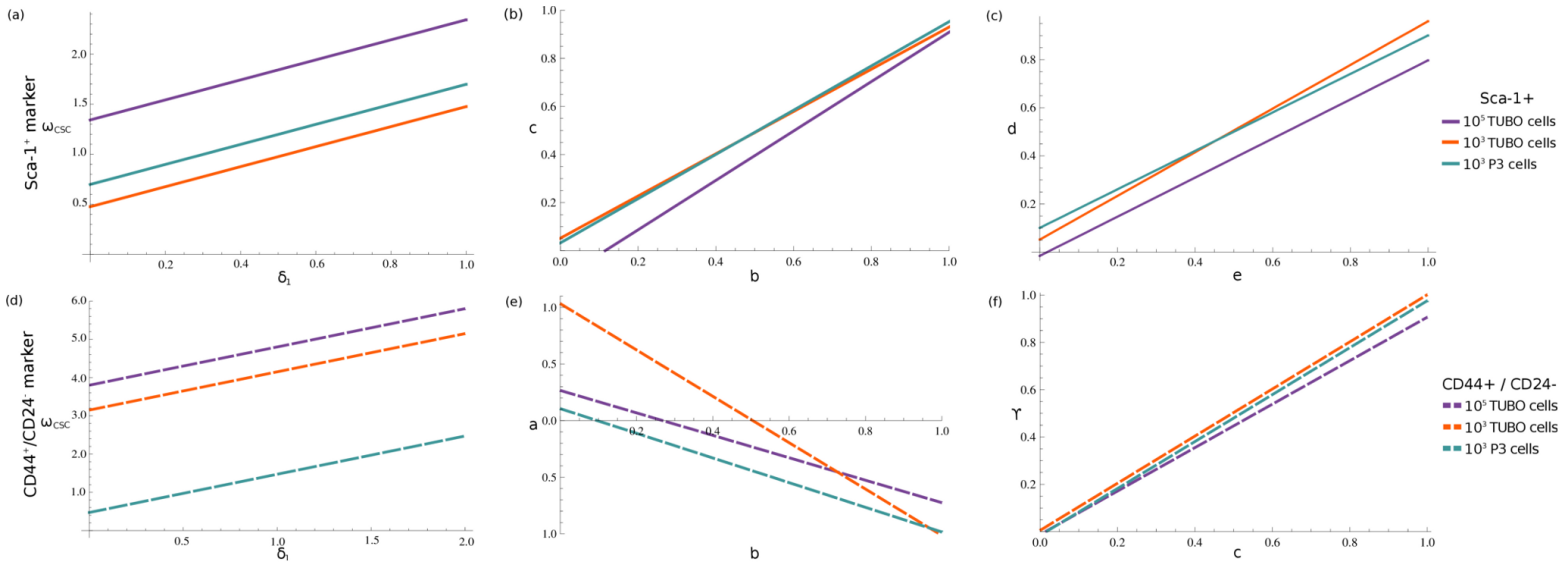
Linear dependencies among parameters. The CSC proliferation rate, 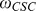, and the CSC death rate, 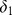, have a mutual linear dependence. Specifically, during initial steps of tumor progression, CSC proliferation is faster than CSC death. Panels (a), (d) show this relationship in the Sca-1^+^ and CD44^+^/CD24^−^ cells scenarios respectively. Other linear dependencies exist among parameters, in both scenarios. Panel (b): the PC_1_ decreasing rate, c, is linearly proportional to the PC_1_ increasing rate, b. Panel (c): linear dependence of the PC_2_ increasing rate, *e*, with respect to their decreasing rate, *d*. Panel (e): liner relationship between the CSC global variation rate, *a*, and the PC_1_ increasing rate, *b*. Panel (f): how the PC_1_ decreasing rate, *c*, is affected by their de-differentiation rate, 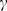. Notice that these linear relationships are valid in all the three injection scenarios. Indeed, parameters show the same qualitative behavior in each panel, independently of their initial conditions.

To extrapolate the relations among original model parameters, the computed best fit-values (Table 1) were assigned to aggregated parameters in system (4). Then, from this set of explicit equations, some dependencies among the original parameters were deduced. More precisely, Figure 4 (panel a) shows how the CSC proliferation rate (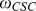) changes with respect to the CSC death rate (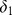). We also observed a linear relationship among the following parameters: CSC differentiation (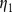), CSC death (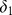), and CSC symmetrical proliferation probability (*P_sy_*), see Figure S4. We may thus assume that there is a trade-off between the transformation/death of CSCs and their proliferation.

We were also interested in discovering hidden relationships among the aggregated parameters. Therefore, a regression analysis was performed on all tuples of values generated by the MLS algorithm and reported in Tables S2–S7 (more details are also provided in Text S1). This analysis emphasized the linear correlations between *b*−*c* and *e*−*d*, shown in Figure 4 (panels b, c). For both correlations, the fitted linear regression models reporting all parameter values are shown in Figure S5. It is possible to observe that the decreasing rate of PC_1_ (*c*) is proportional to the PC_1_ increasing rate (*b*) and a similar behavior exists also between the decreasing (*e*) and increasing (*d*) rates of PCs_2_. Let us note that, when we considered Sca-1^+^ cells obtained by *in vitro* experiments to compute CSC proportions, all these correlations resulted independent of the three experimental scenarios. On the other hand, when CD44^+^/CD24^−^ cells were used, these relations were extrapolated only for 10^3^ P3 experiments. Lastly, linear correlations *b*−*a* and 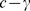 were derived in all three experimental scenarios considering CD44^+^/CD24^−^ proportions, see Figure 4 (panels e, f). It is interesting to notice that the increasing rate of *PC*_1_ (*b*) is correlated with respect to CSC variation (*a*), as well as the PC_1_ decreasing rate (*c*) is correlated with de-differentiation rate (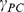) of PCs_1_. The fitted linear regression models with all estimated parameter values are reported in Figure S6.

## Discussion

In this paper we provided an example of how a mathematical model can help to understand a biological phenomenon and to address biological hypotheses. We explored the initial phase of ErbB2^+^ mammary cancer progression in mice focusing on two aspects. First, we investigated the tumorigenic power of the TUBO cell line and of successive mammosphere passages characterizing mechanisms at the basis of tumor progression. Secondly, we performed an accurate analysis on the dependencies among parameters to identify which is the parameter(s) critical to determine an exponential cancer growth. As a consequence, we were able to detect key points in tumor progression that, if altered, can change cancer evolution.

We presented an *essential* model describing the initial phase of breast cancer growth and which gave us the opportunity to reproduce growth volume data obtained from *in vivo* experiments. Among these *in vivo* experiments we selected those corresponding to mice in which the mammary cancer grew exponentially, and we were able to produce a good fit for each initial condition. However, we want to emphasize that our mathematical model can mimic different cancer dynamics. Indeed, the previous analysis of system (2) has revealed how CSCs (mathematically) affect tumor growth since reproduction rate *R*_0_ gives rise to distinct tumor scenarios: exponential cancer growth and tumor extinction. Cell homeostasis will be guaranteed by introducing a feedback mechanism which can maintain a stable equilibrium within tumor cell subpopulations. These scenarios can be investigated in order to identify which are the cellular events that can be perturbed *in vitro* by treatments designed to influence cancer growth. For this purpose, using our model it is possible to investigate at a population level a fine-tuning of model parameters which leads cancer into an extinction condition. An encouraging example of how a computational model combined with experimental data can help to verify how the therapy response influences cell population dynamics is reported in a recent paper by Tyson *et al.* [46]. The authors have showed that erlotinib - an epidermal growth factor receptor inhibitor - is not able to kill tumor cells, but it leads them into a quiescent state or decreases their proliferation rate. Therefore, expressions (6) of possible system behaviors as mathematical equations can give us the possibility to explore both how different drugs work and against which targets, in term of cell events, therapies must be addressed. Note that we further investigated those parameter combinations (best-fit parameters) where CSC reproduction rate (R_0_) is positive, as reported in Table 1.

Previous papers report the crucial role of CSCs to cancer progression [47],[48], but a connection among some of CSC features, i.e. strong self-renewal, resistance to apoptosis, differentiation abilities, and cancer progression, has not been established. Our results suggested which of these features mostly determine cancer growth dynamics, namely those responsible for global CSC and PC variation. Moreover, analyzing parameter values obtained from all runs of the MLS algorithm we discovered some interesting linear correlations among CSC differentiation, CSC death, and CSC symmetrical proliferation probability. This is in accordance with what is observed in many solid tumors or mammosphere models, in which both intrinsic and extrinsic mechanisms known to directly affect CSC symmetric division probability and differentiation or apoptotis have been discovered. These mechanisms, which include p53 mutation or depletion in CSCs [49], and the availability of certain host growth factors - such as EGF and growth-factor-rich niches - can skew division modes in favor of symmetric production of CSCs for up to 85% [50]. Correlations among other parameters were also reported emphasizing a balance among all actions that generate or remove cells within the same subpopulation. In particular, when we imposed cell proportions obtained considering Sca-1^+^ data, these relations were observed in PC_1_ and PC_2_ subpopulations. Otherwise, using data obtained from CD44^+^/CD24^−^ experiments, the trade-off was observed in CSCs. This behavior explains the coexistence of CSCs, PCs, and differentiated cells in the same tumor which, in turn, reflects the cancer heterogeneity that could result from the various differentiation grades of genetically identical cells.

While many markers of CSCs have been described in solid tumors, no specific markers of PCs have been identified yet, and it is probably more accurate to say that a tumor possesses a continuous spectrum of cell types, ranging from CSCs to more differentiated cells [8]. Indeed, the different correlations among parameters that we obtained could be also explained by the fact that, at present time, CSC's markers are specific for the stemness characteristic. Since the identification and characterization of CSC markers is difficult, *CSC marker cocktails* might be more representative of the cancer stem cell biological properties. From our investigations, different linear correlations among parameters have been discovered when different markers have been considered to infer subpopulation proportions. In detail, as summarized in Table 2, it is interesting to note that dependencies involving PC variations are mainly associated with Sca-1 experiments, while correlations on CSC variations have been found considering CD44^+^/CD24^−^ cells.

**Table 2:** Linear parameter dependencies.

	Linear dependencies
**Sca-1^+^ cells**	*b*−*c* (PC_1_)^*^; *e*−*d* (PC_2_)^*^
**CD44^+^/CD24^−^ cells**	*b*−*a*; 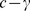 (CSC)^*^

Recapitulation of some hidden relationships among parameters have been extracted using data collected in the fitting process through regression analysis.

**Figure 5:**
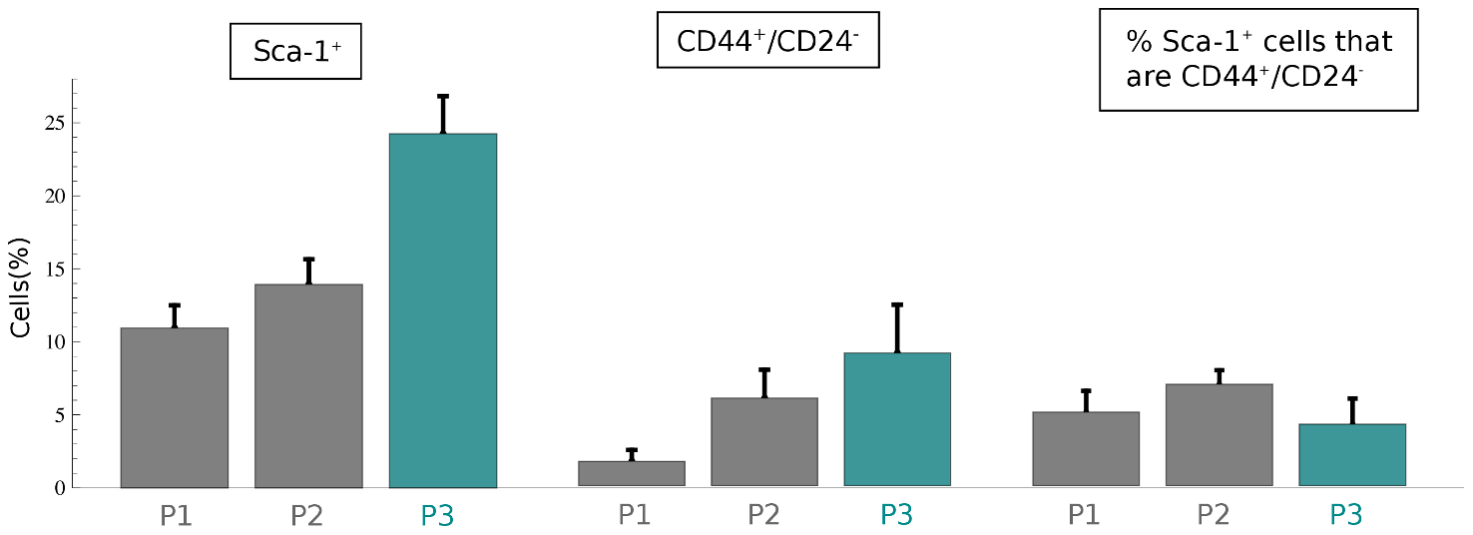
Comparison among markers expression. Sca-1^+^, CD44^+^/CD24^−^ and percentage of Sca-1^+^ positive that are CD44^+^/CD24^−^ histograms reporting the mean ± SEM of positive cells, from six independent experiments.

The model could indicate an association of Sca-1^+^ phenotype with progenitor cells and a connection of CD44^+^/CD24^−^ phenotype with CSCs. These suggestions were considered and preliminarily verified analyzing the percentages of Sca-1^+^/CD44^+^/CD24^−^ cells in all mammosphere passages by FACS analysis. Figure 5 reports a comparison among the percentage of Sca-1^+^, CD44^+^/CD24^−^, and Sca-1^+^/CD44^+^/CD24^−^ cells, in the mammosphere passages. As expected, the amount of CD44^+^/CD24^−^ cells increases. Furthermore, the number of Sca-1^+^ cells is larger than that with CD44^+^/CD24^−^ phenotype. Finally, we found that there is a constant number of Sca-1^+^ cells that are also CD44^+^/CD24^−^. These *in vitro* experiments provided a preliminary evidence that a minimal portion of Sca-1^+^ cells is also CD44^+^/CD24^−^, yielding a first indication that Sca-1^+^ cells might be a marker of PCs. Further investigations will be required to understand if CD44^+^/CD24^−^ phenotype will be lost in Sca-1^+^ cells, as a consequence of the differentiation process of precursor cells. An in-silico validation will be produced fitting volume data using the percentages of CD44^+^/CD24^−^ and Sca-1^+^ cells to indicate the initial concentrations of CSCs and PCs, respectively.

All dynamics considered in our model are related to how cell population vary and cancer progression has been connected to crucial cellular events, as CSC proliferation. Obviously, a deeper characterization of specific cellular events during tumor progression might require the integration of molecular aspects in model. However, we believe that results presented in this paper can be used to facilitate and improve this integration. Hence, in a future work we will combine these results with those of our recent paper [51], in which ErbB2-driven carcinogenesis is described with a multi-level model based on both molecular aspects and cell subpopulation dynamics.

## Supporting Information

Figure S1
**Malthus model fittings.** Each panel considers one of the three initial cell concentrations, and reports a comparison between experimental volumes (points) and the Malthus fit (solid lines). In detail: panel A corresponds to the 10^5^ TUBO cells injection; panel B is related to the 10^3^ TUBO cells experiments, while panel C shows results about the 10^3^ P3 cells case. The quantitative estimation of the growth rates, 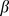, is reported for each case, highlighting the greater tumorigenic potential of P3 cells.(TIFF)

Figure S2 **Subpopulations dynamics and cellular proportions, 10^3^ TUBO cells.** Temporal evolutions of cells subpopulations, panel A, and their proportions in the total tumor mass, panel B, considering the percentage of Sca-1^+^ cells. Temporal evolutions of cells subpopulations, panel C, and their proportions in the total tumor mass, panel D, considering the percentage of CD44^+^/CD24^−^ cells. Once the best-set of parameters is defined, these results are directly derived from the model solution.
(TIFF)

Figure S3
**Possible signs of** 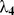 **eigenvalue.** Mutual position in the plane of line *F*(*x*) and: parabola *G*(*x*), panel A; parabola −*G*(*x*), panel B.(TIFF)

Figure S4
**CSCs symmetrical proliferation probability.** In the initial cancer growth phase, the CSCs symmetrical probability (*P_sy_*) expresses a fixed behavior with respect to the CSCs differentiation (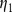), and the CSCs death (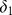). Each panel shows this mutual relationship, considering one of the three initial condition experiments, and one of the two CSCs proportions. In detail: panels A, B and C correspond to the Sca-1^+^ marker scenario having 10^5^ TUBO, 10^3^ TUBO and 10^3^ P3 cells, respectively. On the other side, panels D, E and F are relative to the CD44^+^/CD24^−^ case with 10^5^ TUBO, 10^3^ TUBO and 10^3^ P3 cells injection, respectively.(TIFF)

Figure S5 **Linear regressions among reduced parameters in the Sca-1^+^ marker proportions scenario.** Correlation analysis among parameters-values (obtained from the MLS algorithm) highlights a strong linear correlation between pairs *b*−*c* and *e*−*d*. In each plot scattered parameters values are compared with the corresponding regression line, and the relative correlation coefficient is also reported. Each panel corresponds to correlations between the two pairs, considering one of the three initial conditions. Specifically, reading panels *by columns* it is possible to observe results with respect to the different initial conditions: panels A and D correspond to 10^5^ TUBO cells; panels B and E are relative to 10^3^ TUBO cells; while panels C and F match the 10^3^ P3 cells case. Otherwise, reading panels *by rows*, results are presented considering the parameters-pairs: panels A, B and C are relative to *b*−*c*; while panels D, E and F show *e*−*d* results.
(TIFF)

Figure S6
**Linear regressions among reduced parameters, in the CD44^+^/CD24^−^ marker proportions scenario.** Correlation analysis among parameters-values (obtained from the MLS algorithm) highlights a strong linear correlation between pairs *b*−*a* and 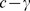. In each plot scattered parameters values are compared with the corresponding regression line, and the relative correlation coefficient is also reported. Each panel corresponds to correlations between the two pairs, considering one of the three initial conditions. Specifically, reading panels *by columns* it is possible to observe results with respect to the different initial conditions: panels A and D correspond to 10^5^ TUBO cells; panels B and E are relative to 10^3^ TUBO cells; while panels C and F match the 10^3^ P3 cells case. Otherwise, reading panels *by rows*, results are presented considering the parameter-pairs: panels A, B and C are relative to *b*−*a*, while panels D, E and F show 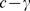 results.(TIFF)

Table S1 **Tumor volume data.** Tumor growth, evaluated as tumor mean diameter (in mm), measured over time in mice injected with 10^5^ TUBO (upper part), 10^3^ TUBO (middle part), and 10^3^ P3 cells (lower part).
(PDF)

Table S2 **Parameters estimation experiments, 10^5^ TUBO cell injection, Sca-1^+^ proportions.** Normalized parameter-values obtained by several runs of the Minimum Least Square algorithm. Within each set of experiments, best fit parameters are highlighted with bold characters. Normalization vectors are reported in Text S1.
(PDF)

Table S3 **Parameters estimation experiments, 10^3^ TUBO cell injection, Sca-1^+^ proportions.** Normalized parameter-values obtained by several runs of the Minimum Least Square algorithm. Within each set of experiments, best fit parameters are highlighted with bold characters. Normalization vectors are reported in Text S1.
(PDF)

Table S4 **Parameters estimation experiments, 10^3^ P3 cells injection, Sca-1^+^ proportions.** Normalized parameter-values obtained by several runs of the Minimum Least Square algorithm. Within each set of experiments, best fit parameters are highlighted with bold characters. Normalization vectors are reported in Text S1.
(PDF)

Table S5 **Parameters estimation experiments, 10^5^ TUBO cells, CD44^+^/CD24^−^ proportions.** Normalized parameter-values obtained by several runs of the Minimum Least Square algorithm. Within each set of experiments, best fit parameters are highlighted with bold characters. Normalization vectors are reported in Text S1.
(PDF)

Table S6 **Parameters estimation experiments, 10^3^ TUBO cells, CD44^+^/CD24^−^ proportions.** Normalized parameter-values obtained by several runs of the Minimum Least Square algorithm. Within each set of experiments, best fit parameters are highlighted with bold characters. Normalization vectors are reported in Text S1.
(PDF)

Table S7 **Parameters estimation experiments, 10^3^ P3 cells, CD44^+^/CD24^−^ proportions.** Normalized parameter-values obtained by several runs of the Minimum Least Square algorithm. Within each set of experiments, best fit parameters are highlighted with bold characters. Normalization vectors are reported in Text S1.
(PDF)

Text S1 **Mathematical analysis.** Description of the mathematical model and its solution; analysis of model eigensystem; results of parameter estimation.
(PDF)
