# Supplementary figures and images for: A Mathematical-Biological Joint Effort to Investigate the Tumor-Initiating Ability of Cancer Stem Cells
Source: PLoS One. 2014 Sep 3;9(9):e106193. doi: 10.1371/journal.pone.0106193 (PMC4153566; doi:10.1371/journal.pone.0106193)

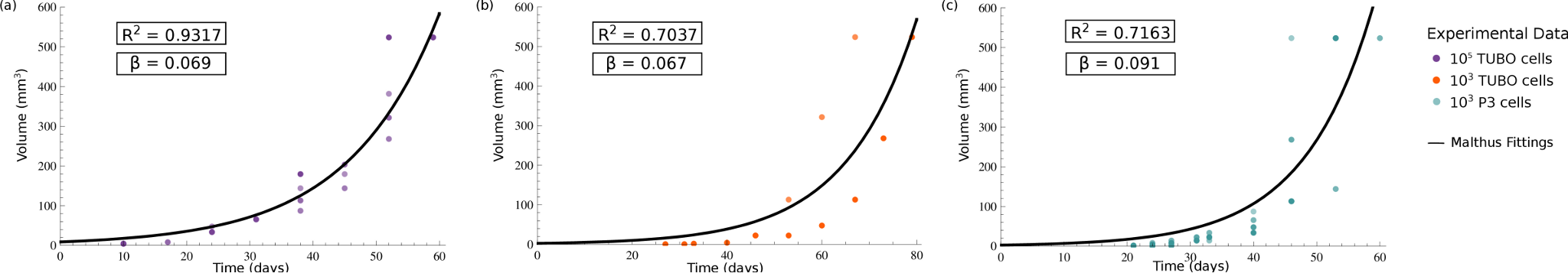

Supplement: Figure S1 — Malthus model fittings. Each panel considers one of the three initial cell concentrations, and reports a comparison between experimental volumes (points) and the Malthus fit (solid lines). In detail: panel A corresponds to the 105 TUBO cells injection; panel B is related to the 103 TUBO cells experiments, while panel C shows results about the 103 P3 cells case. The quantitative estimation of the growth rates, , is reported for each case, highlighting the greater tumorigenic potential of P3 cells. (TIFF) [file pone.0106193.s001.tiff]

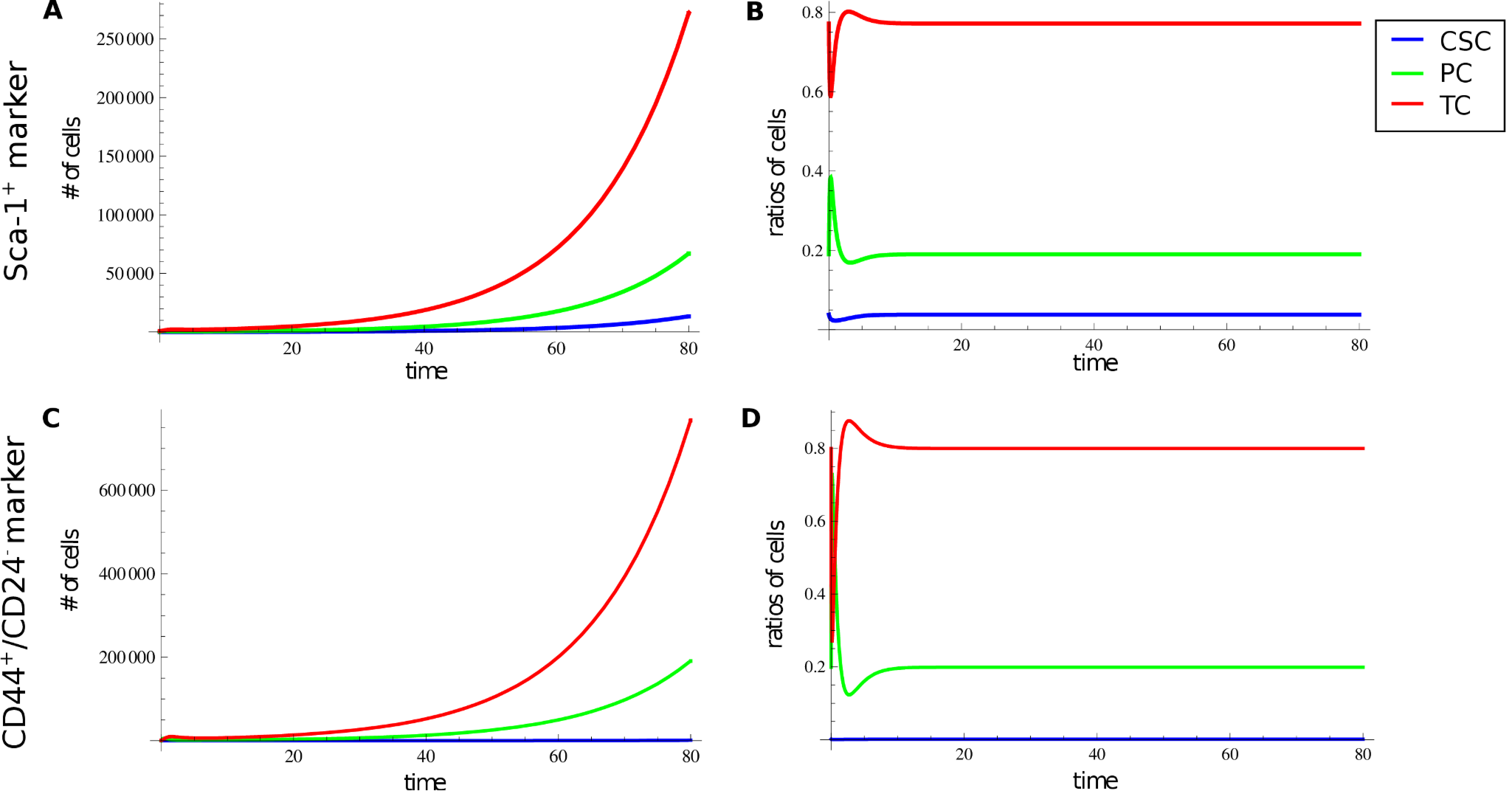

Supplement: Figure S2 — Subpopulations dynamics and cellular proportions, 103 TUBO cells. Temporal evolutions of cells subpopulations, panel A, and their proportions in the total tumor mass, panel B, considering the percentage of Sca-1+ cells. Temporal evolutions of cells subpopulations, panel C, and their proportions in the total tumor mass, panel D, considering the percentage of CD44+/CD24− cells. Once the best-set of parameters is defined, these results are directly derived from the model solution. (TIFF) [file pone.0106193.s002.tiff]

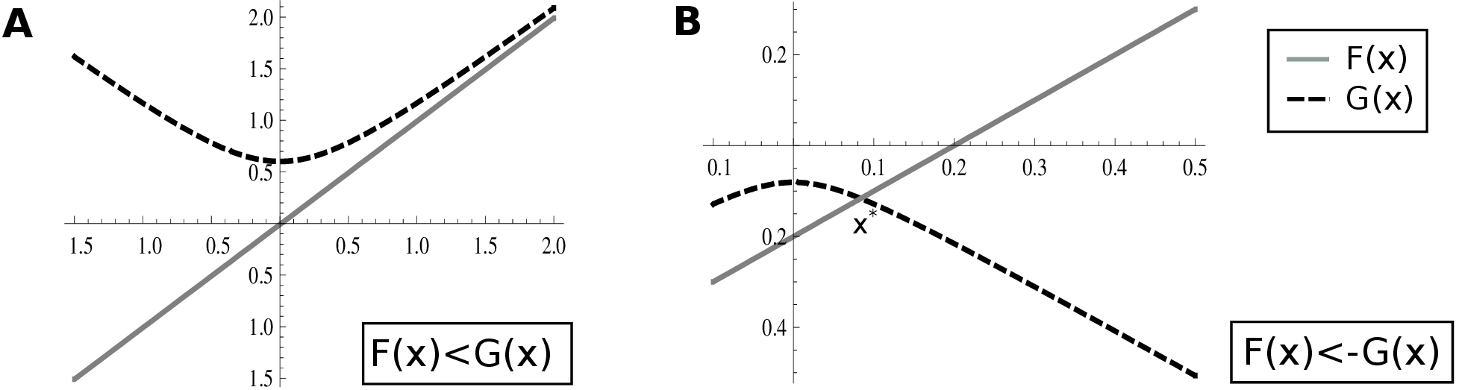

Supplement: Figure S3 — Possible signs of eigenvalue. Mutual position in the plane of line F(x) and: parabola G(x), panel A; parabola −G(x), panel B. (TIFF) [file pone.0106193.s003.tiff]

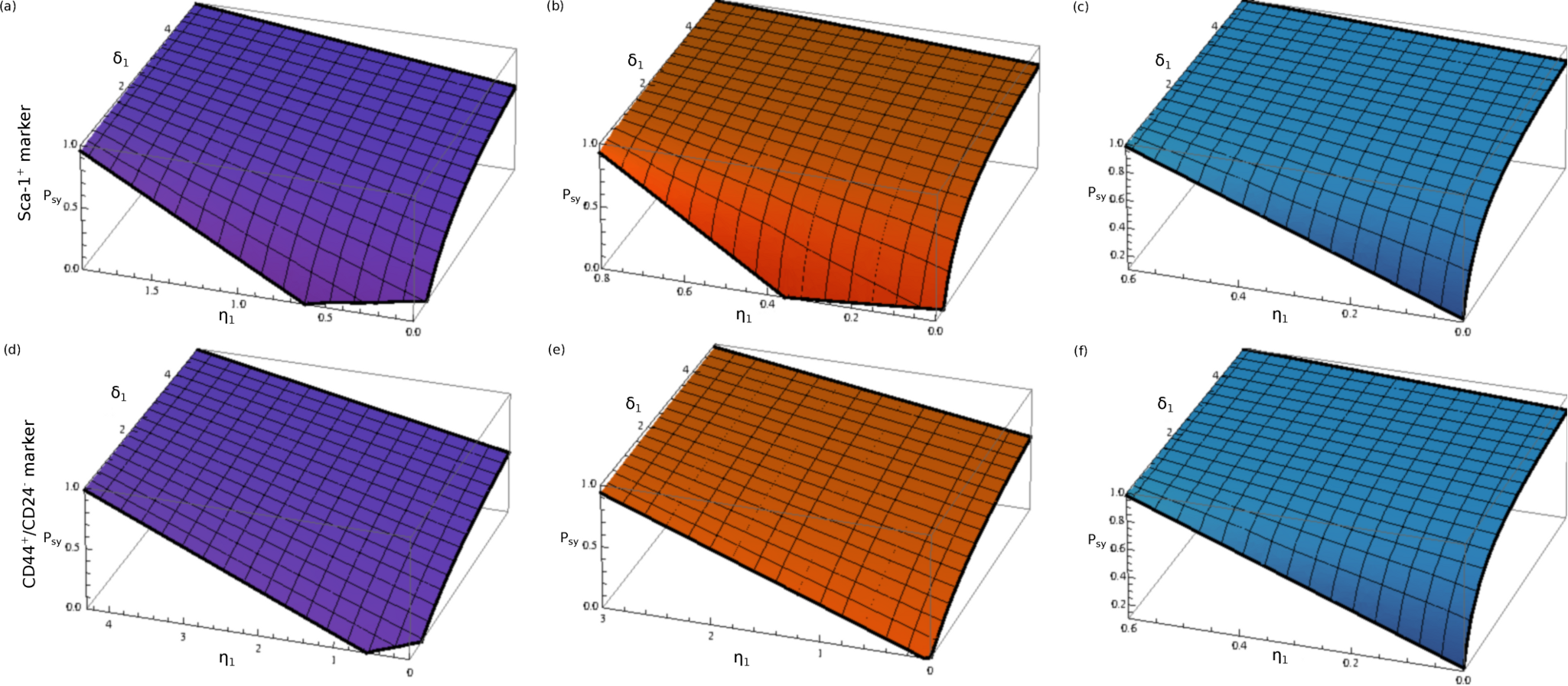

Supplement: Figure S4 — CSCs symmetrical proliferation probability. In the initial cancer growth phase, the CSCs symmetrical probability (Psy) expresses a fixed behavior with respect to the CSCs differentiation (), and the CSCs death (). Each panel shows this mutual relationship, considering one of the three initial condition experiments, and one of the two CSCs proportions. In detail: panels A, B and C correspond to the Sca-1+ marker scenario having 105 TUBO, 103 TUBO and 103 P3 cells, respectively. On the other side, panels D, E and F are relative to the CD44+/CD24− case with 105 TUBO, 103 TUBO and 103 P3 cells injection, respectively. (TIFF) [file pone.0106193.s004.tiff]

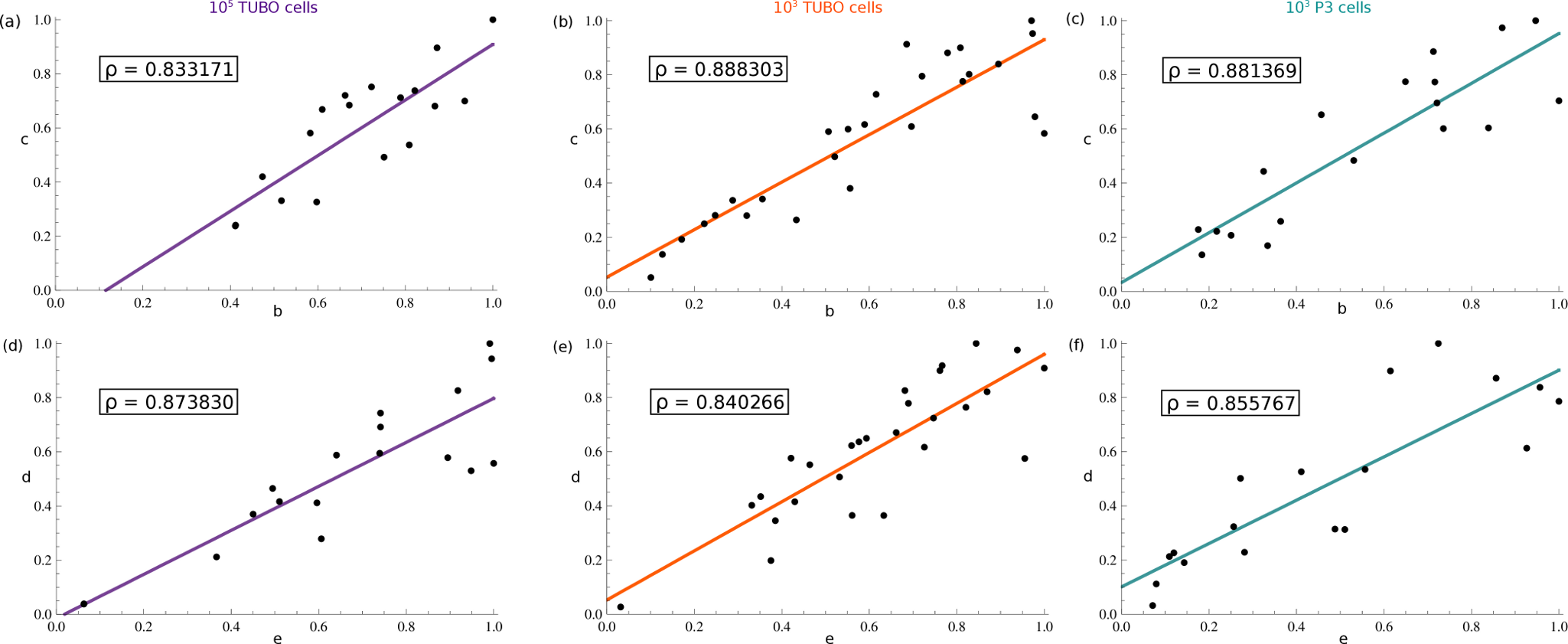

Supplement: Figure S5 — Linear regressions among reduced parameters in the Sca-1+ marker proportions scenario. Correlation analysis among parameters-values (obtained from the MLS algorithm) highlights a strong linear correlation between pairs b−c and e−d. In each plot scattered parameters values are compared with the corresponding regression line, and the relative correlation coefficient is also reported. Each panel corresponds to correlations between the two pairs, considering one of the three initial conditions. Specifically, reading panels by columns it is possible to observe results with respect to the different initial conditions: panels A and D correspond to 105 TUBO cells; panels B and E are relative to 103 TUBO cells; while panels C and F match the 103 P3 cells case. Otherwise, reading panels by rows, results are presented considering the parameters-pairs: panels A, B and C are relative to b−c; while panels D, E and F show e−d results. (TIFF) [file pone.0106193.s005.tiff]

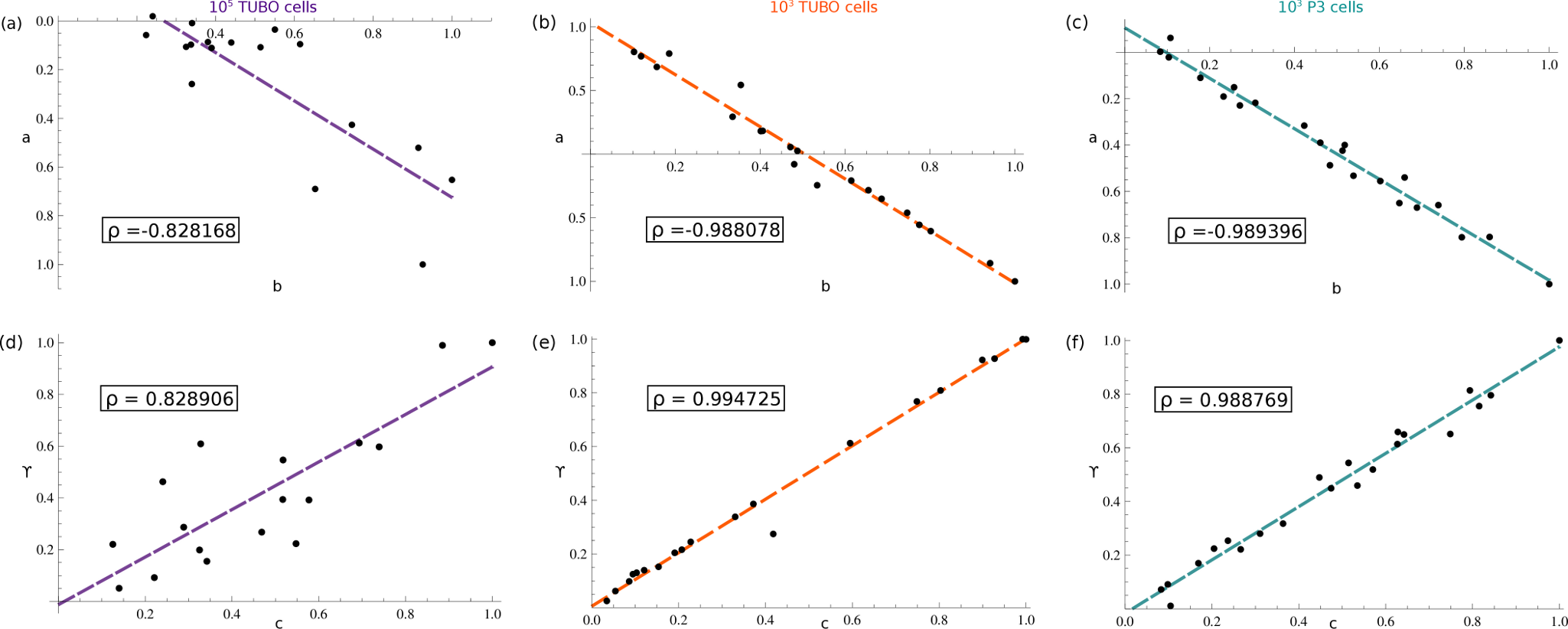

Supplement: Figure S6 — Linear regressions among reduced parameters, in the CD44+/CD24− marker proportions scenario. Correlation analysis among parameters-values (obtained from the MLS algorithm) highlights a strong linear correlation between pairs b−a and . In each plot scattered parameters values are compared with the corresponding regression line, and the relative correlation coefficient is also reported. Each panel corresponds to correlations between the two pairs, considering one of the three initial conditions. Specifically, reading panels by columns it is possible to observe results with respect to the different initial conditions: panels A and D correspond to 105 TUBO cells; panels B and E are relative to 103 TUBO cells; while panels C and F match the 103 P3 cells case. Otherwise, reading panels by rows, results are presented considering the parameter-pairs: panels A, B and C are relative to b−a, while panels D, E and F show results. (TIFF) [file pone.0106193.s006.tiff]
