# Supplementary material for: A Mathematical-Biological Joint Effort to Investigate the Tumor-Initiating Ability of Cancer Stem Cells
Source: PLoS One. 2014 Sep 3;9(9):e106193. doi: 10.1371/journal.pone.0106193 (PMC4153566; doi:10.1371/journal.pone.0106193)
Supplement: Table S1 — Tumor volume data. Tumor growth, evaluated as tumor mean diameter (in mm), measured over time in mice injected with 105 TUBO (upper part), 103 TUBO (middle part), and 103 P3 cells (lower part). (PDF) [file pone.0106193.s007.pdf]

		Time (days)							
		10	17	24	31	38	45	52	59
10^5 TUBO	mouse 1	2	-	4	-	7	-	10	
	mouse 2	2	-	4.5	5	6.5	7	9	10
	mouse 3	2	-	4	5	5.5	6.5	8	10
	mouse 4	2	2.5	-	5	6	7.3	8.5	10

		Time (days)									
		27	31	33	40	46	53	60	67	73	79
10^3 TUBO	mouse 1	1	1	1.5	2	3.5	3.5	4.5	6	8	10
	mouse 2	1	1	1.5	2	3.5	6	8.5	10		

		Time (days)								
		21	24	27	31	33	40	46	53	60
10^3 P3	mouse 1	1.5	2	2.5	3	3.5	4	6	10	
	mouse 2	-	1	1.5	3	3.5	4.5	8	10	
	mouse 3	1.5	2.5	3	3.5	4	5	6	6.5	10
	mouse 4	-	1	1	3	3	4.5	6	10	
	mouse 5	-	-	1.5	3.5	3.5	5.5	10		

Table S1. Tumor volume data. Tumor growth, evaluated as tumor mean diameter (in mm), measured over time in mice injected with 10^5 TUBO (upper part), 10^3 TUBO (middle part), and 10^3 P3 cells (lower part).
