# Supplementary material for: A Mathematical-Biological Joint Effort to Investigate the Tumor-Initiating Ability of Cancer Stem Cells
Source: PLoS One. 2014 Sep 3;9(9):e106193. doi: 10.1371/journal.pone.0106193 (PMC4153566; doi:10.1371/journal.pone.0106193)
Supplement: Table S2 — Parameters estimation experiments, 105 TUBO cell injection, Sca-1+ proportions. Normalized parameter-values obtained by several runs of the Minimum Least Square algorithm. Within each set of experiments, best fit parameters are highlighted with bold characters. Normalization vectors are reported in Text S1. (PDF) [file pone.0106193.s008.pdf]

	**k1**	**b**	**c**	**e**	$\gamma_{\mathbf{PC}}$	$\eta_{\mathbf{3}}$	**a**	**d**	$\delta_{\mathbf{3}}$	$\mathbf{R^2}$
**10^5 TUBO cells, Sca-1$^+$ proportions**	0.9152	0.4111	0.2376	0.0629	0.2995	0.0744	-0.3250	0.0374	0.0005	0.9323
	0.9149	0.4110	0.2386	0.0628	0.2992	0.0743	-0.3229	0.0376	0.0005	0.9323
	0.9137	0.4119	0.2407	0.0631	0.3008	0.0746	-0.3228	0.0380	0.0009	0.9323
	0.8672	0.8089	0.5369	0.3664	0.7012	0.3739	-0.7801	0.2113	0.3039	0.9318
	0.8605	0.5973	0.3262	0.6061	0.6032	0.6150	-0.7972	0.2784	0.5200	0.9318
	0.8135	0.8674	0.6803	0.5960	0.9414	0.5972	-0.9047	0.4114	0.5496	0.9318
	0.8219	0.6718	0.6840	0.4946	0.7326	0.4938	-0.5126	0.4646	0.4646	0.9318
	0.8946	0.7890	0.7116	0.5103	0.6099	0.5109	-0.4779	0.4160	0.4729	0.9318
	0.8105	0.7513	0.4920	0.9486	0.8728	0.9491	-1.0000	0.5296	0.8813	0.9318
	0.8299	0.8725	0.8957	0.7412	0.9213	0.7342	-0.6630	0.6915	0.7263	0.9318
	0.8407	1.0000	1.0000	0.9181	1.0000	0.9073	-0.7495	0.8262	0.9106	0.9318
	**0.9036**	**0.9358**	**0.6992**	**0.8949**	**0.6718**	**0.8922**	**-0.6596**	**0.5783**	**0.8479**	**0.9318**
	0.8143	0.7225	0.7514	0.9953	0.8178	0.9814	-0.5689	0.9433	0.9980	0.9318
	0.8441	0.6099	0.6679	0.9910	0.6260	0.9759	-0.3915	1.0000	1.0000	0.9318
	0.8719	0.8216	0.7378	0.7393	0.6991	0.7349	-0.5621	0.5945	0.7114	0.9318
	0.8755	0.5168	0.3312	1.0000	0.4317	1.0000	-0.4575	0.5572	0.9322	0.9318
	0.8300	0.6622	0.7199	0.7411	0.7162	0.7330	-0.4638	0.7428	0.7319	0.9318
	0.8417	0.5826	0.5806	0.6406	0.5825	0.6364	-0.3998	0.5877	0.6182	0.9318
	1.0000	0.3475	0.1451	0.9339	0.1889	0.9505	-0.2566	0.3296	0.7869	0.9318
	0.9686	0.4731	0.4195	0.4501	0.2635	0.4520	-0.1633	0.3696	0.4102	0.9318

Table S2. Parameters estimation experiments, 10^5 **TUBO cell injection, Sca-1$^+$ proportions**. Normalized parameter-values obtained by several runs of the Minimum Least Square algorithm. Within each set of experiments, best fit parameters are highlighted with bold characters. Normalization vectors are reported in Text S1.
