# Supplementary material for: A Mathematical-Biological Joint Effort to Investigate the Tumor-Initiating Ability of Cancer Stem Cells
Source: PLoS One. 2014 Sep 3;9(9):e106193. doi: 10.1371/journal.pone.0106193 (PMC4153566; doi:10.1371/journal.pone.0106193)
Supplement: Table S4 — Parameters estimation experiments, 103 P3 cells injection, Sca-1+ proportions. Normalized parameter-values obtained by several runs of the Minimum Least Square algorithm. Within each set of experiments, best fit parameters are highlighted with bold characters. Normalization vectors are reported in Text S1. (PDF) [file pone.0106193.s010.pdf]

	k1	b	c	e	γ_{PC}	η_3	a	d	δ_3	R^2
**10^3 P3 cells, Sca-1$^+$ proportions**	0.9965	0.9467	1.0000	0.0794	0.1479	0.0933	0.0829	0.1114	0.0431	0.7164
	**0.9992**	**0.3248**	**0.4430**	**0.1093**	**0.0212**	**0.1174**	**0.1893**	**0.2123**	**0.0882**	**0.7164**
	0.9975	0.7127	0.8858	0.6147	0.0865	0.5847	0.1430	0.8979	0.6926	0.7164
	1.0000	0.8389	0.6037	0.4880	0.1453	0.5112	0.0321	0.3140	0.4279	0.7164
	0.9885	0.3638	0.2583	0.9264	0.2858	0.9471	-0.1239	0.6128	0.8726	0.7164
	0.9310	0.1757	0.2284	0.1196	0.3239	0.1269	0.0021	0.2261	0.1005	0.7164
	0.9885	0.5308	0.4836	0.5567	0.1891	0.5537	0.0287	0.5346	0.5644	0.7164
	0.9885	0.1838	0.1347	0.2811	0.0865	0.2956	0.1122	0.2282	0.2435	0.7164
	0.9885	0.2505	0.2069	0.9565	0.0940	0.9457	0.1116	0.8378	0.9845	0.7164
	0.9885	0.4567	0.6524	0.2719	0.1847	0.2645	0.0931	0.5012	0.2912	0.7163
	0.9884	0.7163	0.7736	0.2564	0.1680	0.2590	0.0703	0.3228	0.2496	0.7163
	0.9885	0.7358	0.6013	1.0000	0.1930	1.0000	0.0032	0.7857	1.0000	0.7163
	0.9885	0.7211	0.6957	0.8563	0.0892	0.8353	0.1240	0.8713	0.9110	0.7163
	0.9125	0.6491	0.7745	0.7242	0.6593	0.6887	-0.2665	1.0000	0.8163	0.7136
	0.9125	1.0000	0.7040	0.5104	1.0000	0.5369	-1.0000	0.3124	0.4417	0.7096
	0.9125	0.8701	0.9734	0.4109	0.0972	0.4023	0.1282	0.5257	0.4334	0.7086

Table S4. Parameters estimation experiments, 10^3 **P3 cells injection, Sca-1$^+$ proportions**. Normalized parameter-values obtained by several runs of the Minimum Least Square algorithm. Within each set of experiments, best fit parameters are highlighted with bold characters. Normalization vectors are reported in Text S1.
