# Supplementary material for: A Mathematical-Biological Joint Effort to Investigate the Tumor-Initiating Ability of Cancer Stem Cells
Source: PLoS One. 2014 Sep 3;9(9):e106193. doi: 10.1371/journal.pone.0106193 (PMC4153566; doi:10.1371/journal.pone.0106193)
Supplement: Table S6 — Parameters estimation experiments, 103 TUBO cells, CD44+/CD24− proportions. Normalized parameter-values obtained by several runs of the Minimum Least Square algorithm. Within each set of experiments, best fit parameters are highlighted with bold characters. Normalization vectors are reported in Text S1. (PDF) [file pone.0106193.s012.pdf]

	k1	b	c	e	γ_{PC}	η_3	a	d	δ_3	R^2
10^3 TUBO cells, $CD44^+/CD24^-$ proportions	0.7024	0.6549	0.9274	0.7765	0.9272	0.7778	-0.2843	0.9107	0.7709	0.7038
	0.7029	0.7461	1.0000	0.9034	0.9991	0.9035	-0.4618	1.0000	0.9029	0.7038
	0.7024	0.9416	0.9922	0.7914	1.0000	0.7928	-0.8577	0.6888	0.7853	0.7038
	0.6941	0.7746	0.8992	0.8479	0.9226	0.8486	-0.5558	0.8138	0.8445	0.7038
	0.7024	0.6858	0.8032	0.7610	0.8088	0.7626	-0.3514	0.7390	0.7541	0.7038
	0.7024	1.0000	0.7484	0.8735	0.7678	0.8746	-1.0000	0.5399	0.8687	0.7038
	0.3769	0.3688	0.3180	0.6125	0.8325	0.6158	-0.8462	0.4475	0.5986	0.7038
	0.7024	0.8015	0.5948	1.0000	0.6126	1.0000	-0.6055	0.6144	1.0000	0.7038
	0.7025	0.4710	0.3721	0.5024	0.3861	0.5068	0.0552	0.3354	0.4838	0.7038
	0.7181	0.6145	0.3302	0.8086	0.3382	0.8108	-0.2090	0.3646	0.7990	0.7038
	0.8923	0.3540	0.4179	0.6490	0.2741	0.6517	0.5436	0.6447	0.6376	0.7038
	**0.3979**	**0.3817**	**0.3477**	**0.3583**	**0.8399**	**0.3639**	**-0.7685**	**0.2787**	**0.3345**	**0.7038**
	0.7029	0.4012	0.2279	0.5777	0.2447	0.5817	0.1808	0.2809	0.5604	0.7038
	0.7132	0.4057	0.1909	0.6522	0.2042	0.6559	0.1822	0.2640	0.6363	0.7038
	0.7026	0.1561	0.2072	0.4023	0.2158	0.4072	0.6849	0.4653	0.3811	0.7038
	0.7310	0.1024	0.1536	0.4612	0.1517	0.4655	0.8029	0.6124	0.4427	0.7038
	0.7026	0.4799	0.1033	0.6149	0.1297	0.6209	-0.0800	0.1175	0.5897	0.7038
	0.7025	0.5340	0.0945	0.5970	0.1242	0.6037	-0.2454	0.0941	0.5682	0.7038
	0.7618	0.4874	0.0860	0.5995	0.0977	0.6062	0.0255	0.0952	0.5709	0.7038
	0.4206	0.3322	0.0552	0.4134	0.1922	0.4209	-0.9026	0.0662	0.3819	0.7038
	0.7036	0.3348	0.1208	0.5129	0.1393	0.5181	0.2933	0.1643	0.4906	0.7038
	0.7412	0.1191	0.0543	0.6260	0.0615	0.6298	0.7688	0.2806	0.6099	0.7038
	1.0000	0.1854	0.0346	0.8064	0.0249	0.8113	0.7902	0.1592	0.7859	0.7038

Table S6. Parameters estimation experiments, 10^3 **TUBO cells,** $CD44^+/CD24^-$ **proportions**. Normalized parameter-values obtained by several runs of the Minimum Least Square algorithm. Within each set of experiments, best fit parameters are highlighted with bold characters. Normalization vectors are reported in Text S1.
