# Supplementary material for: A Mathematical-Biological Joint Effort to Investigate the Tumor-Initiating Ability of Cancer Stem Cells
Source: PLoS One. 2014 Sep 3;9(9):e106193. doi: 10.1371/journal.pone.0106193 (PMC4153566; doi:10.1371/journal.pone.0106193)
Supplement: Text S1 — Mathematical analysis. Description of the mathematical model and its solution; analysis of model eigensystem; results of parameter estimation. (PDF) [file pone.0106193.s014.pdf]

Supporting Text

Mathematical Model

Model solution

To simplify the notation, we express the initial value problem described by Equations (2) and (3) using the following matrix form

$$\begin{cases} X(t)' = & AX(t) \\ X(0) = & X_0, \end{cases} \tag{s.1}$$

where A is the 4×4 matrix containing the biological coefficients of system (2), and $X(t) = (x_1(t),\ldots,x_4(t)) = (N_{CSC}(t), N_{PC_1}(t),\ N_{PC_2}(t), N_{TC}(t))$ is the vector representing cancer cells subpopulations as functions of time.
It is well known that the solution of this type of Cauchy problem (s.1) is given by

$$X(t) = \sum_{i=1}^{4} c_i e^{\lambda_i t} W_i, \tag{s.2}$$

where λ_i $(i = 1, \ldots, 4)$ are the eigenvalues of A, and $W_1, \ldots, W_4$ are the corresponding eigenvectors [1]. Therefore, if a set of initial cell subpopulations is specified, the model (s.1) can be analytically solved to obtain the cardinalities of each cell subpopulations at any point in time. Moreover, from the general expression of $X(t)$ given in (s.2), it is evident that eigenvalues and eigenvectors determine the temporal evolution of this linear dynamic system and its stability as well [1].
Three cases can occur theoretically:

(i) asymptotic stability, i.e. $X(t) \to 0$, when all the eigenvalues have negative real parts;

(ii) asymptotic instability, i.e. $X(t) \to \infty$, when at least one eigenvalue has a positive real part;

(iii) simple stability, i.e. $X(t) \to \Sigma$, when at least one eigenvalue has a null real part, and the others have negative real parts.

Due to these considerations, within the spectrum $\sigma(A) = \{\lambda : \exists v \neq 0, \text{ such that } Av = \lambda v\}$ of matrix A, there is one eigenvalue that drives the temporal evolution of the system. It is called growth constant, $\alpha(A)$[1], and it corresponds to the eigenvalue with the maximum real part. More precisely, considering λ_s as the growth constant (i.e. $\lambda_s = \alpha(A)$) and W_s as the corresponding eigenvector[2], the role of $\alpha(A)$ is clear from the following limits:

$$\lim_{t\to\infty} \frac{x_j(t+\Delta t)}{x_j(t)} = e^{\lambda_s \Delta t}, \qquad \lim_{t\to\infty} r_j(t) = \frac{x_j(t)}{x_1(t)} = \frac{w_{js}}{w_{1s}}, \qquad j = 1, \ldots, 4, \tag{s.3}$$

which define the growth rate of the system, and its growth direction, respectively.

System eigenvalues and eigenvectors

Using aggregate parameters (4), matrix A becomes:

$$A = \begin{pmatrix} a & \gamma_{PC} & 0 & 0 \\ b & -c & 0 & 0 \\ 0 & d & -e & 0 \\ 0 & 0 & \eta_3 & -\delta_3 \end{pmatrix}, \tag{s.4}$$

[1] $\alpha(A) = max_i \mathcal{R}e(\lambda_i)$.
[2] $W_s = [w_{1s}, w_{2s}, w_{3s}, w_{4s}]^T$

thus decreasing the set of parameters that must be inferred form data: a, b, c, d, e, γ_{PC}, η_3, and δ_3. Let us note that A is a Metzler[3] matrix [2] and that, introducing

$$A' = \begin{pmatrix} a & \gamma_{PC} \\ b & -c \end{pmatrix}, \quad B' = \begin{pmatrix} 0 & 0 \\ 0 & 0 \end{pmatrix}, \quad C' = \begin{pmatrix} 0 & d \\ 0 & 0 \end{pmatrix}, \quad D' = \begin{pmatrix} -e & 0 \\ \eta_3 & -\delta_3, \end{pmatrix},$$

A can be written in the following block form

$$A = \begin{pmatrix} A' & B' \\ C' & D' \end{pmatrix}.$$

Therefore, eigenvalues of matrix A correspond to those of its submatrices A' and D'[4]. Moreover, being D' a triangular matrix, its eigenvalues are its diagonal values, i.e. $-e$, $-\delta_3$, which are negative numbers. Consequently, model solution $X(t)$ is mostly characterized by the subsystem associated with submatrix A', which describes CSC and PC_1 variations.

More generally, eigenvalues of matrix A correspond to the following parameter combinations:

$$\lambda_1 = -\delta_3, \;\; \lambda_2 = -e, \;\; \lambda_{3/4} = \frac{1}{2}\left(a - c \mp \sqrt{(a+c)^2 + 4b\gamma_{PC}}\right). \tag{s.5}$$

As showed before, λ_1 and λ_2 are negative numbers, while signs of λ_3 and λ_4 have to be discussed. For this purpose, we used a graphical approach that allowed us to analyze how line $F(x) = x - 2c$ and parabola $G(x) = \sqrt{x^2 + 4b\gamma_{PC}}$ move in the plane according to changes in parameter values[5]. Using this methodology we derived some algebraic conditions that constrain λ_3 and λ_4 to be negative. Specifically, there are two cases to distinguish between:

(i) $F(x) < G(x)$, to study the sign of λ_3;

(ii) $F(x) < -G(x)$, to study the sign of λ_4.

In details:

(i) $F(x) < G(x)$. Since the intercept of $F(x)$ is below the vertex of $G(x)$, namely $-2c < 2\sqrt{b\gamma_{PC}}$, we have that $F(x) < G(x)$ for all x. Therefore λ_3 is negative, and its sign does not depend on the parameter values. See Figure S3 panel A.

(ii) $F(x) < -G(x)$. The intersection point between $F(x)$ and $G(x)$ is $x^* = (c^2 - b\gamma_{PC})/c$. Therefore, the sign of λ_4 depends on inequality $a - c < (c^2 - b\gamma_{PC})/c$. See Figure S3 panel B.

To summarize, λ_4 is the only eigenvalue whose sign can change, while the other eigenvalues are negative. Therefore, the stability of system (2) is controlled by λ_4 and, given experimental data of Figure 1 - panel a, the growth constant $\alpha(A)$ corresponds to λ_4. Its corresponding eigenvector W_4 is defined as:

$$W_4 = \begin{pmatrix} w_{14} \\ w_{24} \\ w_{34} \\ w_{44} \end{pmatrix} = \begin{pmatrix} \theta\beta\sigma\delta\nu\epsilon \\ \theta\beta\sigma\delta \\ \theta\beta \\ 1 \end{pmatrix}, \tag{s.6}$$

[3]Metzler matrices are the important class of matrices that have non-negative off-main-diagonal elements.

[4]$det(A) = det(A')det(D')$.

[5]$x = a + c$.

where

$$\theta = \frac{1}{2\eta_3} \quad \beta = a - c + 2d_3 + \sqrt{(a+c)^2 + 4b\gamma_{PC}}$$
$$\sigma = \frac{1}{2d} \quad \delta = a - c + 2e + \sqrt{(a+c)^2 + 4b\gamma_{PC}}$$
$$\nu = \frac{1}{2b} \quad \epsilon = a + c + \sqrt{(a+c)^2 + 4b\gamma_{PC}}.$$

Lastly, knowing the analytic solution (s.2) of model (2), it is easy to express conditions (5) on subpopulation proportions as:

$$lim_{t\to\infty}\frac{N_j(t)}{N(t)} = \frac{w_{js}}{w_{1s} + w_{2s} + w_{3s} + w_{4s}} = k_j^{[i]}, \quad \text{(s.7)}$$

where $W_s = [w_{1s}, w_{2s}, w_{3s}, w_{4s}]^T$ is the eigenvector corresponding to λ_s, i.e. to λ_4, and j= CSCs,PCs,TCs.

Parameter estimation

Tables S2, S3, S4, S5, S6, and S7 report results obtained in the data fitting process, i.e. the output generated in the several runs of MLS algorithm[6]. Results are organized as follows:

- each table contains parameter values obtained from the fitting of tumor data assuming specific initial conditions. More precisely, each table reports results relative to a specific type of cell injection and to fixed proportions among subpopulations.
- Values reported in tables are normalized with respect to maximum values found for each set of experiments. The normalization vector $[\overline{k1}, \overline{b}, \overline{c}, \overline{e}, \overline{\gamma_{PC}}, \overline{\eta_3}, \overline{a}, \overline{\delta_3}, \overline{d_3}]$ is hence evaluated for each initial condition, i.e. considering injections of 10^5 TUBO, 10^3 TUBO and 10^3 P3 cells, and considering subpopulation proportions suggested by Sca-1^+ and $CD44^+/CD24^-$ cells. Normalization vectors are reported below.

Normalization vector for Sca-1$^+$ experiments

- $[16.08, 2.09, 3.20, 5.75, 1.21, 4.85, 0.94, 44.06, 0.96]_{10^5\text{TUBO}}$;
- $[456.74, 2.35, 3.65, 5.91, 2.23, 4.92, 1.70, 42.40, 0.98]_{10^3\text{TUBO}}$;
- $[657.56, 1.89, 4.91, 4.63, 1.02, 3.34, 0.45, 9.20, 1.29,\ 0.72]_{10^3\text{P3}}$.

Normalization vector for CD44$^+$/CD24$^-$ experiments

- $[4.89, 4.37, 3.03, 4.54, 0.41, 3.71, 0.87, 1078.29, 0.84]_{10^5\text{TUBO}}$;
- $[355.86, 8.39, 5.97, 7.46, 0.10, 6.04, 0.07, 1299.35, 1.42]_{10^3\text{TUBO}}$;
- $[647.77, 3.66, 9.49, 10.85, 3.51, 9.50, 1.25, 37.97,\ 1.36]_{10^3\text{P3}}$.

Discovering relationships among reduced parameters

In order to extract dependencies among model parameters, we analyzed all tuples originated by MLS algorithm, i.e. values reported in Tables S2, S3, S4, S5, S6, and S7. In detail, we performed a linear correlation analysis among parameters and, when possible, we evaluated also the corresponding regression model. We discovered strong linear correlations within the pairs $b - c$, $e - d$ in Sca-1^+ proportion experiments, and between parameters $b - a$ and $c - \gamma$ in $CD44^+$ $CD24^-$ ones. More precisely, correlation coefficient (ρ) was greater than 0.8 in each evaluation. Regression results are reported in Figures S5 and S6.

[6] The MLS algo minimizes the difference between experimental values and the fitted ones provided by a model.

References

1. Pierre NVT (1992) Dynamical systems. An introduction with application in economics and biology: Springer-Verlag.
2. Mitkowski W, (2008) Dynamical properties of Metzler systems. B Pol Acad Sci 54: 309-312.
